# Consumption of fruits, vegetables, and legumes are associated with overweight/obesity in the middle- and old-aged Chongqing residents: A case-control study

**DOI:** 10.1097/MD.0000000000029749

**Published:** 2022-07-08

**Authors:** E Wu, Juntao Ni, Wei Zhou, Leiying You, Lin Tao, Tian Xie

**Affiliations:** a School of Pharmacy, Hangzhou Normal University, Hangzhou, Zhejiang, China and Key Laboratory of Elemene Class Anti-Cancer Chinese Medicines; Engineering Laboratory of Development and Application of Traditional Chinese Medicines; Collaborative Innovation Center of Traditional Chinese Medicines of Zhejiang Province, Hangzhou Normal University, Hangzhou, Zhejiang, China; b Women’s Hospital School of medicine Zhejiang University, Hangzhou, Zhejiang, China; c The Second People’s Hospital of Banan District, Chongqing, China.

**Keywords:** behaviors, dietary habits, overweight/obesity

## Abstract

This study aimed to investigate the association of dietary habits with the risk of overweight/obesity among middle-and-old-aged Chongqing residents and also to examine the joint effects of behavioral lifestyles, dietary habits, and overweight/obesity.

In this case-control study, age (±3 years), sex, and time of physical exercise matched 979 overweight/obesity residents, and 979 normal weight residents were recruited.

A validated questionnaire was used to collect participants’ information. Conditional logistic regression analysis was performed to determine the adjusted odds ratios (ORs) and 95% CIs of dietary habits and lifestyles associated with overweight/obesity risk.

Overweight/obesity was defined as body mass index (BMI) ≥ 24 kg·m^−2^, and normal weight was defined as 18.5 ≤ BMI < 24 kg·m^−2^.

The multivariate-adjusted models showed the weekly intake frequency of fruits 0–1 (day/week) (OR = 1.79, 95% CI = 1.04–3.10), and legumes 0–1 (day/week) (OR = 2.45, 95% CI = 1.28–4.67), as well as the weekly intake percentage of vegetables ≥ 15% (OR = 2.44, 95% CI = 1.04–5.71) were associated with a higher risk of overweight/obesity. Besides, there were joint effects of lifestyles (smoking or drinking) and dietary habits on overweight/obesity risk (*P* for interaction < 0.05).

The consumption of vegetables, fruits, legumes, and the joint effects of behavioral habits (smoking or drinking) may modify the risk of being overweight/obese. It is essential to consume fruits and legumes at least 2 days/week, quit smoking, and stop consuming alcohol to avoid overweight/obesity among middle-aged and elderly people in Chongqing, China.

## 1. Introduction

Overweight/obesity is defined by WHO as the abnormal or excessive accumulation of fat.^[1]^ It has emerged as one of the major public health issues and has become more and more common in the past century, with the rapid growth of the global economy and the improvement of people’s living standards, coupled with unreasonable diet.^[2]^ In the past decades, China has presented a rapid increase in the prevalence of overweight/obesity.^[3]^ In 2018, China was globally ranked as having the largest number of overweight/obese people.^[4]^ According to a recent national survey, over half of the Chinese adults had either overweight or obese, which contributes to 11.1% of noncommunicable disease-related deaths,^[5]^ making overweight/obesity emerge as a major public health concern and healthcare system challenge.

Overweight/obesity results from a combination of factors. Although genetic conditions may be related to obesity to some extent, dietary habits are reported to be significantly associated with the risk of overweight/obesity.^[6,7]^ Some dietary habits such as meal frequency or the variety of consumed food may be related to body weight, BMI, or other indicators of overweight/obesity. The current data point that dietary habits, such as high intake of fried food, junk food, sugared beverages, and processed meat, are attributed to increased risk of overweight/obesity.^[8]^ Notably, fruits and vegetables, especially high green leafy vegetables, have low energy density and high dietary fiber content and are rich in phytochemicals such as terpenoids and polyphenols,^[9]^ which may play a key role in preventing overweight and obesity. Additionally, intake of legumes was thought to be used to prevent and manage obesity due to their richness in plant protein.^[10]^ However, the role of certain foods, such as fruit, vegetables, and legumes, in overweight/obesity remains inconsonant and controversial; the consumption of these foods and its association with weight gain in individuals in different regions remain uncertain.^[11,12]^

Besides dietary habits, the increasing rates of obesity are largely attributed to lifestyles; people presenting with a variety of behaviors have been linked to an individual’s weight gain, including drinking, smoking, and so on.^[13]^ Living a sedentary lifestyle has also been regarded as a risk factor for overweight/obesity, and 150 to 200 minutes of physical exercise per week is considered effective in preventing overweight/obesity. However, current data on the impact of dietary habits and lifestyle on obesity/overweight among middle-and-old-aged Chinese residents are limited. Therefore, considering the severe comorbidities and rising medical costs associated with overweight/obesity, it is necessary to explore the potential association between dietary habits and lifestyles in obesity/overweight middle- and old-age people for early prevention and treatment of overweight/obesity.

Therefore, this study aimed to examine the association between dietary habits and overweight/obesity among middle- and old-aged Chongqing Chinese residents and also to investigate the interactions between lifestyles, dietary habits, and overweight/obesity.

## 2. Materials and Methods

### 2.1. Study population

We collected data on 3194 middle-and-old-aged Chongqing China participants through a multistage stratified cluster random sampling method from September 6, 2019, and May 1, 2020. The inclusion criteria were: (1) local residents who had lived in Chongqing for at least 1 year, and (2) the exact age was 45 to 74 years (subject to the date of birth on the ID card). The exclusion criteria were: (1) pregnant and breastfeeding women and (2) individuals diagnosed with cancer, hypothyroidism, hyperthyroidism, and dwarfism. We further excluded participants with missing values (n = 932), and BMI <18.5 kg·m^−2^ (n = 159). According to the proposal from the working group on obesity in China (WGOC), the definition of overweight in China is 24 kg·m^−2^≤ BMI < 28 kg·m^−2^, obesity is BMI ≥ 28 kg·m^−2^, and normal weight is 18.5 kg·m^−2^≤ BMI < 24 kg·m^−2^.^[14]^ We, therefore, defined overweight/obesity as BMI ≥ 24 kg·m^−2^. Participants who were overweight/obese were defined as cases, while the controls with normal weight were matched with sex, age (±3 years), and time of physical exercise. Of the remaining 2103 participants, 979 cases and 979 controls were successfully matched (Supplementary material Figure S1 http://links.lww.com/MD/G790). According to the 1:1 case-control study, sample size calculation formula is as follows^[15]^:

$$\begin{array}{l} \begin{array}{l} \begin{array}{l} m={\left[z_{\alpha }/2+z_{p}\sqrt{p(1-p)}\right]}^{2}/{\left(\;p-1\right)}_{2}\\ p=OR/(1+OR)\approx RR/(1+RR)\\ M\approx m/(p_{0}q_{1}+p_{1}q_{0}) \end{array} \end{array} \end{array}$$

where α = 0.01 (2-sided), β = 0.10, power = 0.9, assuming that the exposure ratio of drinking in the control group was 5%, namely *p*_0_ = 0.05, and the estimated OR was 2. The estimated minimum sample size was 207 pairs. This study planned to use 979 pairs.

### 2.2. Data collection

To calculate the BMI, participants’ weight and height were measured by trained nurses, and the BMI was calculated by dividing the weight (Kg) by height (M) squared. A questionnaire was administered by trained nurses through face-to-face interviews to collect participants’ information. Sociodemographic information factors, including age (years), sex (male, female), birthplace (Chongqing, others), marriage status (single, married, divorced, widowed), educational level (primary school and below, junior middle school, high school and above) was obtained. Behavior lifestyle factors included physical exercise (did you exercise more than 3 times a week and each time over 30 minutes in the past year: yes, no), smoking (did you smoke more than 1 cigarette per day for more than 6 months in the past year: yes, no), and alcohol consumption (did you drink nearly every day in the past year: yes, no) were obtained. Data on disease history previously diagnosed in medical institutions, including hypertension, diabetes mellitus (DM), and hyperlipidemia, were also obtained.

Information on dietary habits, including the approximate weekly frequency and percentage consumption of vegetables, fruits, and legumes in the past year, was gathered. To avoid the extreme groups with limited cases, we divided FWI (frequency of weekly intake) into 4 categories: every day, 4 to 6 days/week, 2 to 3 days/week, and 0 to 1 day/week. PWI (Percentage of Weekly Intake) refers to the percentage of the weight of the specific type of food in the total diet.

### 2.3. Statistical analysis

The continuous variables (normally distributed) were presented as mean ± standard (SD), and categorical variables were presented in frequencies (%). The restricted cubic spline (RCS) with 3 knots was used to explore the dose-response correlations. The conditional logistic regression for adjusted odds ratio (OR) with 95% confidence interval (CI) was used to analyze the strength of the relationship between diet, lifestyle, and overweight/obesity; we included age, sex, physical exercise, marriage status, and education in model 1 and additionally adjusted for disease history of hypertension, DM, and hyperlipidemia in model 2. Overweight/obesity and normal weight were considered outcomes, while dietary habits, smoking, and drinking were considered the main exposure variables. Crossover analyses were used to analyze the joint effects between diet behavior. We also tested the term beta in the entire model to examine interactions.

Sensitivity analysis was conducted by redefining BMI ≥23 kg·m^−2^ as cases, and 18.5 kg·m^−2^ ≤ BMI < 23 kg·m^−2^ as controls. According to the proposal from the WHO, the definition of overweight in Asian populations is as follows: normal weight means 18.5 kg·m^−2^ ≤ BMI < 23 kg·m^−2^, overweight means 23 kg·m^−2^ ≤ BMI < 27.5 kg·m^−2^, and obesity means BMI ≥ 27.5 kg·m^−2^.^[16,17]^

Statistical analyses were performed using SAS version 9.1 (SAS Institute, Cary, NC), and graphs were plotted using *R* (version 4.1.3). Two-sided *P* < 0.05 was considered statistically significant.

### 2.4. Ethics approval and consent to participate

This study was approved by the Research Ethics Committees of Hangzhou Normal University, and all the study participants signed the informed consent (Ethics Approval Number 2020-001).

## 3. Results

### 3.1. Basic characteristics of the study participants

The general characteristics of the 979 overweight/obesity and 979 normal weight participants were shown in Table 1. The mean age of the case group and the control group were 56.7 and 56.5 years, respectively. Compared with participants with normal weight, those with overweight/obesity were more likely to be smokers, drinkers, not married; had a lower weekly intake of legumes and higher weekly intake percentage of vegetables; and were more likely to report a disease history of hypertension, DM, and hyperlipidemia.

**Table 1.** Basic characteristics of the study participants with and without overweight/obesity.

Variable	Cases (n = 979)	Controls (n = 979)
Age (yr, mean ± SD)	56.7 ± 7.9	56.5 ± 7.7
Male, n (%)*	321(32.8)	321(32.8)
Smoke, n (%)*	173(17.7)	117(12.0)
Drink, n (%)*	87(8.9)	59(6.0)
Hypertension, n (%)*	142(14.5)	56(5.7)
Diabetes, n (%)*	76(7.8)	37(3.8)
Hyperlipidemia, n (%)*	76(7.8)	44(4.5)
Exercise, n (%)*	436(44.5)	438(44.7)
Marriage status, n (%)		
Married	921(94.1)	927(94.7)
Divorce/single	26(2.7)	20(2.0)
Widowed	32(3.3)	32(3.3)
Education, n (%)		
Primary and below	361(36.9)	346(35.3)
Junior middle	391(39.9)	376(38.4)
High and above	227(23.2)	257(26.3)
Vegetables PWI, n (%)	17.7 ± 5.1	17.3 ± 5.1
≥15%	803(82.0)	771(78.8)
<15%	168(17.2)	189(19.3)
<10%	8(0.8)	19(1.9)
Fruit FWI, n (%)		
7 (d/W)	442(45.1)	484(49.4)
4–6 (d/W)	306(31.3)	307(31.4)
2–3 (d/W)	192(19.6)	165(16.9)
0–1 (d/W)	39(4.0)	23(2.3)
Fruit PWI, n (%)	13.5 ± 5.7	13.3 ± 5.2
>15%	277(28.3)	252(25.7)
≤15%	278(28.4)	316(32.3)
≤10%	305(31.2)	282(28.8)
≤5%	119(12.2)	129(13.2)
Legumes FWI, n (%)		
7 (d/W)	16(1.6)	31(3.2)
4–6 (d/W)	180(18.4)	211(21.6)
2–3 (d/W)	555(56.7)	553(56.5)
0–1 (d/W)	228(23.3)	184(18.8)
Legumes PWI, n (%)	7.1 ± 3.5	7.6 ± 4.0
>15%	5(0.5)	15(1.5)
≤15%	67(6.8)	99(10.1)
≤10%	382(39.0)	365(37.3)
≤5%	525(53.6)	500(51.1)

Abbreviations: FWI = frequency of weekly intake; d/W = days/Week; PWI = percentage of weekly intake.

* The column percentages for the binary variable sum to 100, and present the percentages of the “yes” option.

### 3.2. Association between dietary, lifestyle factors, and overweight/obesity risk

**Figure 1.**
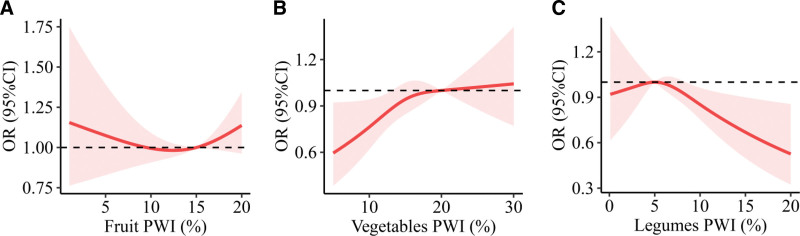
Multivariable adjusted dose-response associations between weekly intake percentage of vegetables, fruit and legumes and overweight/obesity risk. (a) Fruit, (b) Vegetables, (c) Legumes. Adjusted for age, sex, physical exercise, marriage status, and education. All *P* for nonlinearity > 0.05.

Figure 1 presents a dose-response association between the weekly intake percentage of vegetables, fruits, and legumes and overweight/obesity risk. The U-shaped association between the weekly intake percentage of fruit and overweight/obesity risk was observed. When fruit PWI was between 10% and 15%, the risk of overweight/obesity was at a lower level. The RCS model also revealed that the risk of overweight/obesity increased as vegetables’ PWI raised and leveled off at 15% to 20%. Regarding legumes, when legumes’ PWI was over 10%, the risk of overweight/obesity decreased as legumes’ PWI increased. Therefore, we used 5%, 10%, 15% as the cut points to categorize the PWI of vegetables, fruits, and legumes (Table 1).

**Figure 2.**
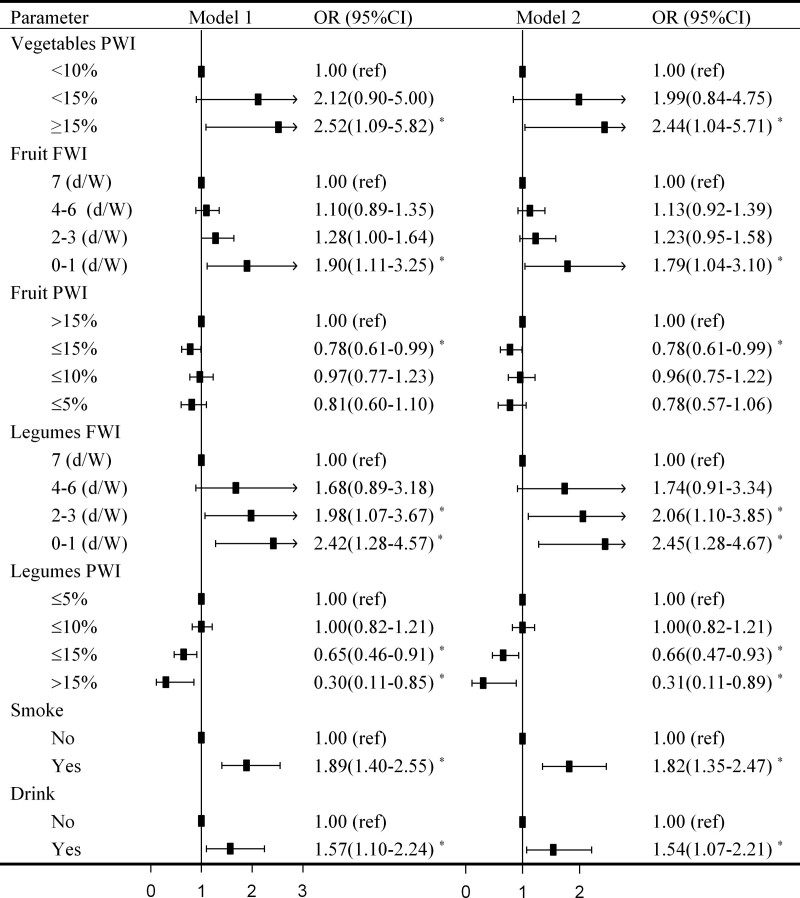
OR (95% CI) of overweight/obesity risk according to potential factors. Abbreviations: FWI, frequency of weekly intake; PWI, percentage of weekly intake; d/W, days/Week; Model 1 adjusted for sex, age, physical exercise, marriage status, and education, Model 2 adjusted for model 1 and disease history of hypertension, DM, hyperlipidemia.

After full adjustment in model 2, we found that PWI of legumes >10%, and 10% <fruits ≤15% were associated with a lower risk of overweight/obesity (OR < 1, *P* < 0.05) (Fig. 2). Moreover, vegetables ≥15%, fruit FWI 0-1 (d/W), legumes FWI 0-1 (d/W), smoking and drinking were associated with increased overweight/obesity risk, the respective ORs (95% CIs) were 2.44 (1.04–5.71), 1.79 (1.04–3.10), 2.45 (1.28–4.67), 1.82 (1.35–2.47), and 1.54 (1.07–2.21). The results are consistent with Figure 1.

### 3.3. Association between the number of high-risk factors and overweight/obesity risk

We further classified vegetables PWI ≥ 15%, fruits FWI 0-1 (d/W), fruits PWI ≤10% or >15%, legumes FWI 0-1 (d/W), legumes PWI ≤5%, smoking, and drinking as high-risk factors on overweight/obesity. As shown in Table 2, the multivariate-adjusted models indicated that the risk of overweight/obesity increased as the number of high-risk factors increased. Individuals with 6 or more risk factors had 6.15 higher odds (95%CI: 2.25–16.83) of being overweight/obese, compared with individuals with 0 or 1 high-risk factor (*P* for trend < 0.001).

**Table 2.** OR (95% CI) of overweight/obesity risk according to the number of high-risk factors.

NHRF	Controls (n = 979)	Cases (n = 979)	OR (95%CI)	*P*-value	*P* for trend
≤1	207(21.1)	164(16.8)	1.00(ref)		<0.001
2	386(39.4)	344(35.1)	1.12(0.87–1.45)	0.373	
3	236(24.1)	266(27.2)	1.42(1.07–1.87)	0.014	
4	124(12.7)	143(14.6)	1.50(1.08–2.07)	0.016	
5	21(2.1)	39(4.0)	2.28(1.26–4.09)	0.006	
≥6	5(0.5)	23(2.3)	6.15(2.25–16.83)	<0.001	

NHRF: Number of high-risk factors.

NHRF contained 7 high-risk factors, including vegetables PWI ≥ 15%, fruit FWI 0-1 (d/W), fruit PWI >15% or ≤10%, legumes FWI 0-1 (d/W), legumes PWI ≤5%, smoking, and drinking. Models adjusted for sex, age, physical exercise, marriage status, education, disease history of hypertension, DM, and hyperlipidemia.

### 3.4. Joint effects between dietary habits and lifestyles on overweight/obesity risk

**Figure 3.**
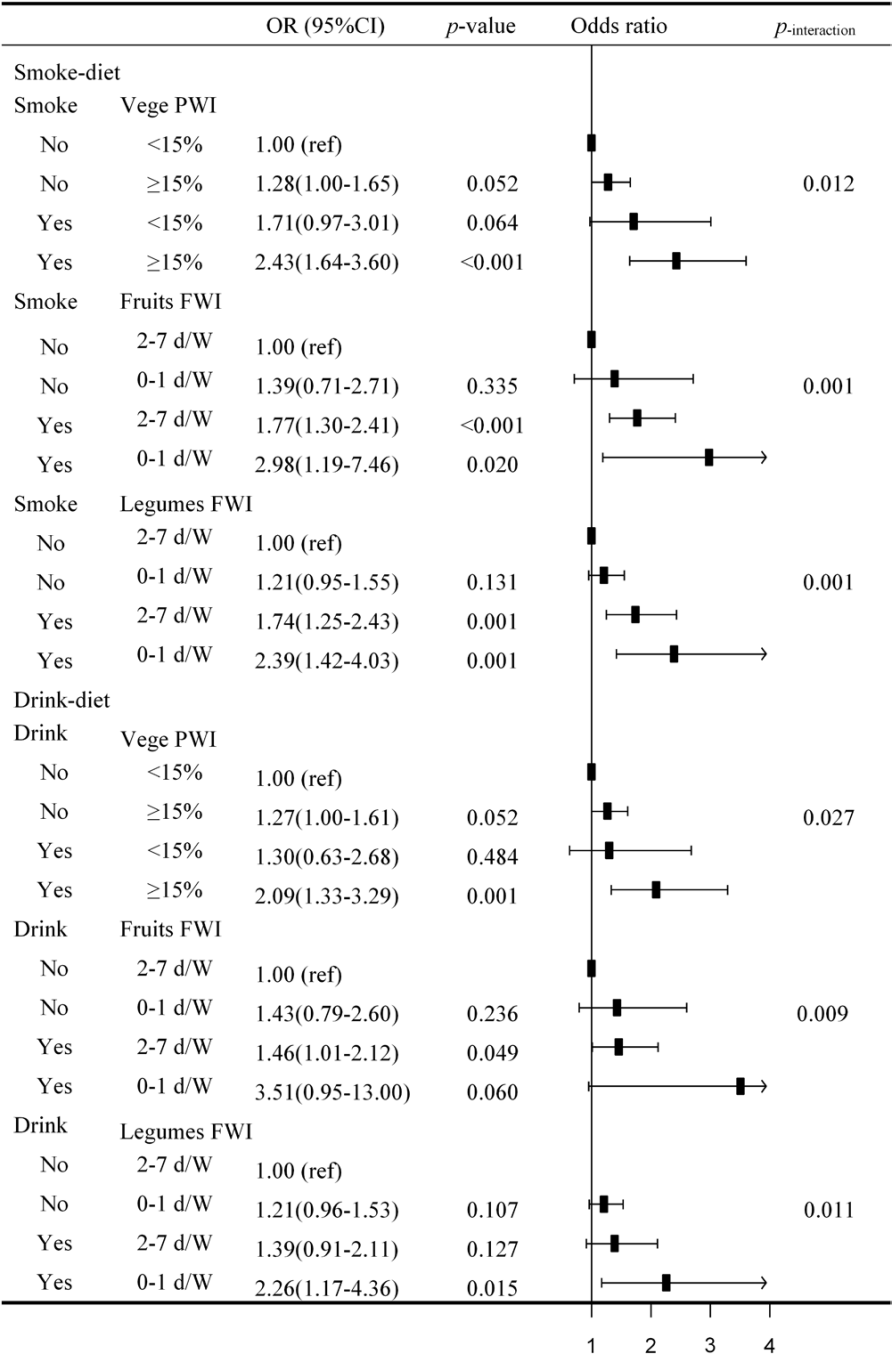
OR (95% CI) of overweight/obesity risk according to dietary habits and behavioral lifestyles interactions. Abbreviation: FWI, Frequency of Weekly Intake; PWI, Percentage of Weekly Intake; d/W, days/Week; Vege, Vegetables; *, *P* < 0.05. Conditional logistic regression analysis adjusted for sex, age, physical exercise, marriage status, education, disease history of hypertension, DM, and hyperlipidemia.

Crossover analysis revealed an interaction between lifestyle factors, such as drinking, smoking, and dietary habits for overweight/obesity risk (Fig. 3). The association of vegetables PWI ≥ 15%, fruit FWI 0-1 (d/W), and legumes FWI 0-1 (d/W) with the risk of overweight/obesity were consistently stronger in participants with smoking (*P* for interaction < 0.05). Stronger associations for PWI ≥ 15%, fruit FWI 0-1 (d/W), and legumes FWI 0-1 (d/W) were also identified in participants with drinking behavior (*P* for interaction < 0.05).

### 3.5. Sensitivity analyses

In sensitivity analyses, similar results were observed after redefining the BMI ≥23 kg·m^−2^ as cases. The RCS models presented a similar trend of results (Supplementary material Figure S2 http://links.lww.com/MD/G791). The multivariate-adjusted models confirmed fruit FWI and legumes FWI 0-1 (d/W) as high-risk factors for overweight/obesity (HR < 1, *P* < 0.05) (Supplementary material Figure S3 http://links.lww.com/MD/G792). Interactions between dietary habits and lifestyles were also observed (Supplementary material Figure S4 http://links.lww.com/MD/G793).

## 4. Discussion

The association between dietary habits and other factors and overweight/obesity in middle-and-old-aged Chongqing residents was investigated. In this study, we found significant differences between overweight/obesity cases and controls in the consumption of fruits, vegetables, and legumes. Compared with daily consumption of fruits, vegetables, and legumes, the consumption frequency of fruit and legumes in zero or 1 day a week was considered a high-risk factor for overweight/obesity. Weekly intake of vegetables <10%, 10% <fruits ≤15%, and legumes ≥15% were associated with decreased risk of overweight/obesity. Some scholars have reported that fruits, vegetables, and legumes may be treated as a healthy diet.^[18,19]^ However, the relationship between fruits, vegetables, legumes intake, and weight change remains uncertain, and limited literature is available, especially for middle-aged and elderly people.^[20]^

Some American and Japanese studies have reported that the intake of vegetables and fruits negatively correlates with weight changes.^[21,22]^ In contrast, a previous study from Europe found that the intake of vegetables and fruits had no significant difference in weight changes, and this was contrary to our analysis.^[23]^ The difference may be due to the living environment of the 2 populations, or it may be due to racial differences. We innovatively found that the consumption of fruits and vegetables within an appropriate range may help to control weight in Chinese. Therefore, middle-aged and elderly people should consume fruit and legumes at least 2 days per week, a weekly intake of legumes over 10%, 10% <fruit ≤15%, and vegetables <15% to control weight.

The consumption of fruits and vegetables in moderation may help lose weight due to a reduction in total energy intake since the higher water contained in fruits and vegetables may increase satiety.^[24]^ Moreover, there is little fat content in fruits and vegetables, which are also rich in vitamins and minerals, which may decrease adipocyte differentiation and proliferation.^[25,26]^ Additionally, fruits and vegetables contain various phytochemicals, such as polyphenols, terpenoids, and organosulfur, which affect the expression level of certain genes related to antiobesity function, and play an important role in adipose tissue growth and differentiation, apoptosis of adipocytes, and lipid and energy metabolism.^[27]^ For example, the down-regulation of oxidative stress can increase lipolysis to control hyperlipidemia, thereby reducing adipogenesis and increasing fat cell apoptosis to prevent overweight/obesity.^[28]^ There was no significant difference in the association between the frequency of vegetable consumption and overweight/obesity. This may be due to more than 90% of the residents eating vegetables every day in both cases and controls. Excessive consumption of vegetables (≥ 15% per week) may increase the risk of overweight/obesity since some vegetables contain high levels of carbohydrates. When the body consumes too many carbohydrates, which cannot be absorbed within a short period, the excess carbohydrates accumulate in the body and are converted into fat, increasing the risk of overweight/obesity.

Legumes are low energy-dense foods that are nutritionally rich.^[29]^ Many scholars have found that regular consumption of legumes may be beneficial for preventing and managing overweight/obesity.^[30,31]^ However, it is necessary to understand the intake and frequency of legumes required for potential health benefits. In a study conducted in Chile, people who ate legumes less than once a week were highly inclined to become overweight, and these findings were consistent with our study.^[32]^ This study recommended that middle-aged and elderly people consume legumes at least 2 days a week, and the weekly intake is above 10%. There are three possible mechanisms through which the consumption of adequate amounts of legumes may help in weight control. First, most legumes are low in fat content and are rich in vitamin B and other essential nutrients and minerals, which reduce the risk of being overweight/obese. Second, legumes are rich in protein, amylose, and dietary fiber. The interaction of protein and starch further decreases digestibility, and dietary fiber increases satiety and improves weight management.^[33]^ Moreover, legumes also have high levels of oligosaccharides, which are associated with flatulence. High concentrations of oligosaccharides contribute to increased production of propionate, which is reported to reduce serum cholesterol levels and induce satiety.^[34]^ Lastly, legumes contain a variety of bioactive compounds, such as phenolic compounds, oligosaccharides, and enzyme inhibitors, which were thought to help in weight control. These bioactive compounds influence the expression levels of low-density lipoprotein, inhibit cholesterol synthesis, reduce oxidative stress, control hyperlipidemia by enhancing lipolysis and decreasing lipogenesis, and decreasing the fat mass by reducing adipogenesis and increasing adipoapoptosis.

The effect of fruits, vegetables, and legumes on overweight/obesity is moderate, emphasizing the need to combine with other interventions for better weight control. Some studies attribute the high prevalence of certain diseases such as diabetes and hypertension to an increased incidence of overweight/obesity.^[35]^ Previous studies reported that the consumption of fruits and vegetables might help decrease the risk of T2DM and hypertension.^[36]^ Legumes are effective in controlling diabetes and hyperlipidemia due to their “slow-release carbohydrates” properties.^[37]^

In this study, we classified smoking, drinking, fruits FWI 0-1 (d/W), legumes FWI 0-1 (d/W), and vegetables PWI ≥ 15% as high-risk factors. We discovered interactions between behavior, lifestyle, and dietary habits to overweight/obesity. Therefore, this study recommends a healthy diet for middle-aged and elderly people, which includes consumption of fruits and legumes at least 2 days per week and a weekly intake of vegetables of <15%. It is also applicable to populations with hypertension, DM, hyperlipidemia, and people with smoking and drinking habits.

There are some limitations to this study. First, we obtained dietary and lifestyle information through self-reports, although we trained nurses before collecting data. Measurement errors were still inevitable. Second, even though we have adjusted sociodemographic, medical history, etc, in models to investigate the association between dietary and overweight/obesity, unmeasured confounders could still exist. Third, the results of this study are only applicable to middle-aged and elderly people, as children, adolescents, pregnant and breastfeeding women, and residents of other provinces in China were excluded. Follow-up studies with larger sample sizes are necessary. Forth, as a cross-sectional study, this study cannot explain causality. It would be better to carry out a cohort study to supplement the results, and randomized controlled trials are necessary for the future. Finally, the research capacity of stratified analysis was limited by the sample size, requiring more subjects to participate in future research.

## 5. Conclusions

In conclusion, this study suggests that the differences in vegetables, fruits, and legumes intake may modify the risk of overweight/obesity. These differences still exist, especially in people with different behavioral habits (smoking or drinking). In subsequent studies, the experimental subjects will be followed, and dietary information will be collected from a larger population to validate the results of this study.

## Author contributions

E Wu and Juntao Ni performed the statistical analysis and wrote the manuscript. Tian Xie and Lin Tao designed the study and revised the manuscript. Leiying You was responsible for quality control and conducted the experiments. Wei Zhou was responsible for data collection. All authors have read and agreed to the published version of the manuscript.

## Acknowledgment

Not applicable.

## Supplementary Material


