# Supplementary figures and images for: Consumption of fruits, vegetables, and legumes are associated with overweight/obesity in the middle- and old-aged Chongqing residents: A case-control study
Source: Medicine (Baltimore). 2022 Jul 8;101(27):e29749. doi: 10.1097/MD.0000000000029749 (PMC9259125; doi:10.1097/MD.0000000000029749)

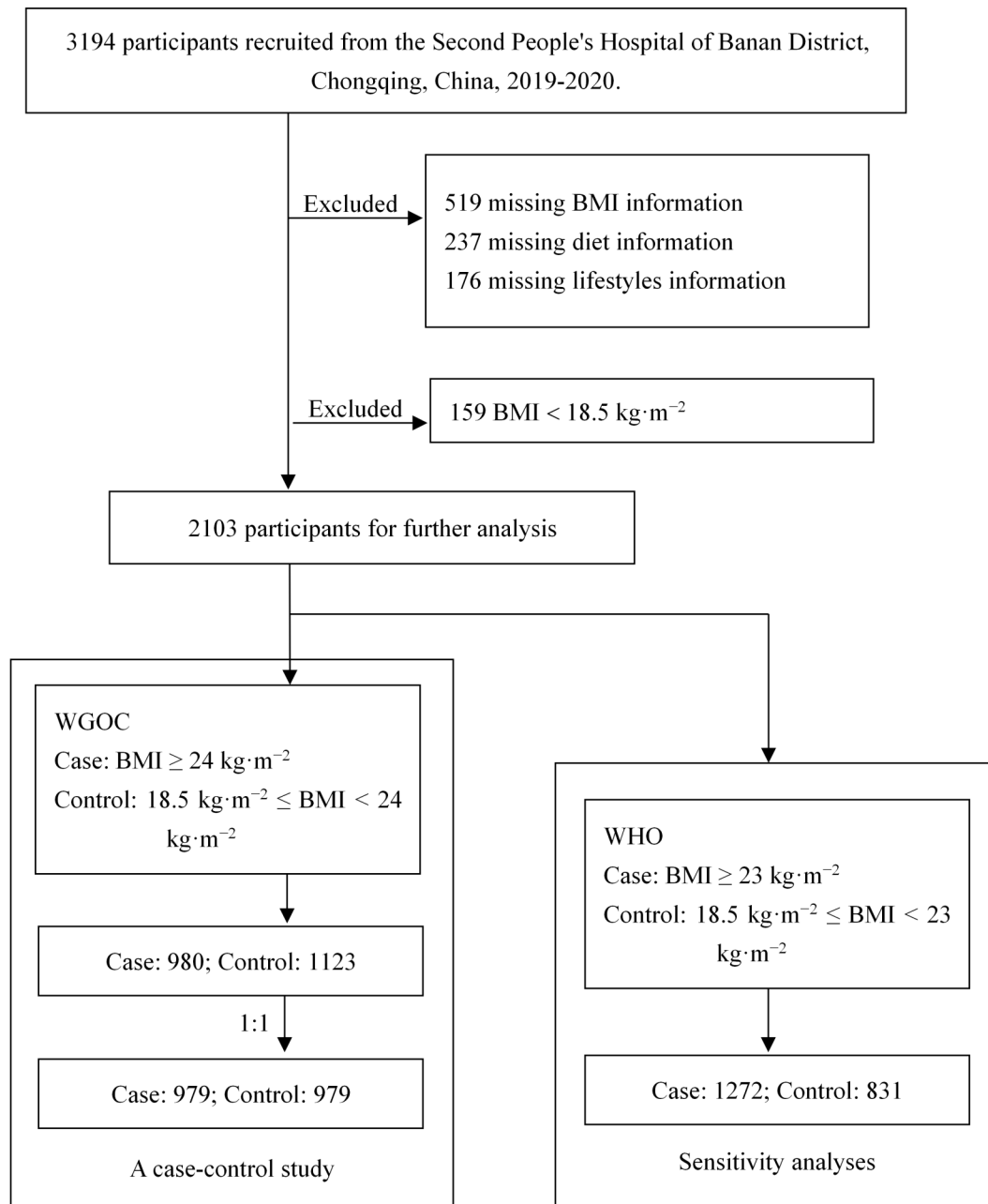

**Figure S1.** Flow diagram

Supplement: Supplementary file 1 [file medi-101-e29749-s001.pdf]
