# Supplementary material for: Consumption of fruits, vegetables, and legumes are associated with overweight/obesity in the middle- and old-aged Chongqing residents: A case-control study
Source: Medicine (Baltimore). 2022 Jul 8;101(27):e29749. doi: 10.1097/MD.0000000000029749 (PMC9259125; doi:10.1097/MD.0000000000029749)
Supplement: Supplementary file 2 [file medi-101-e29749-s002.pdf]

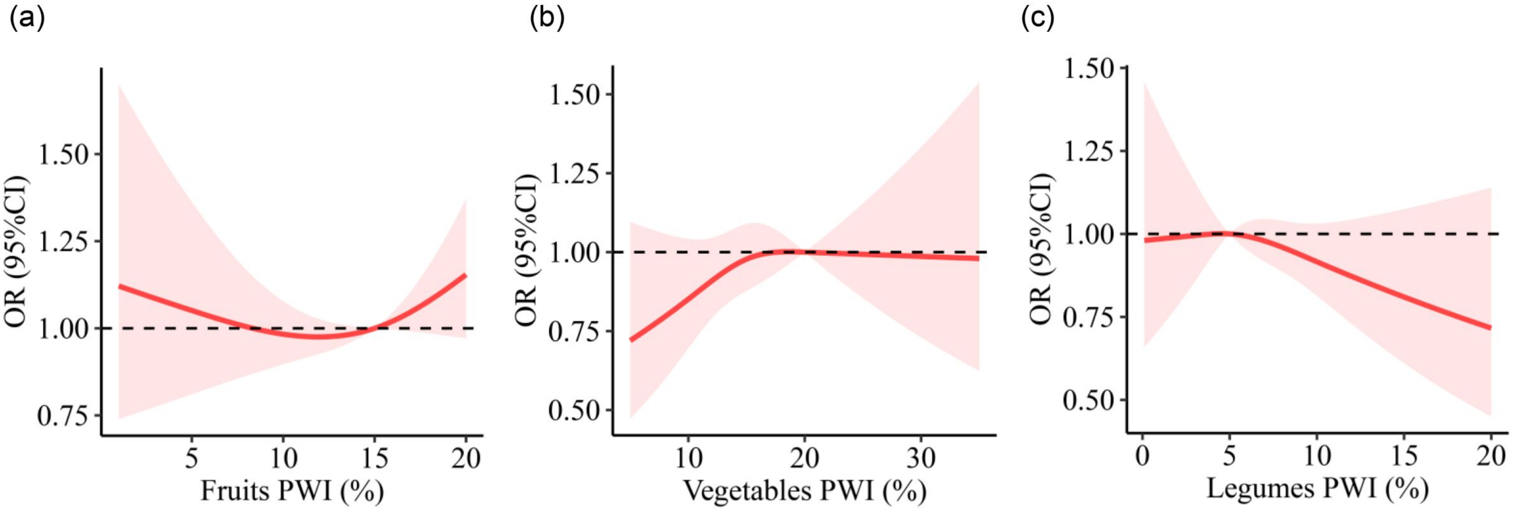

Figure S2. Multivariable adjusted dose-response associations between weekly intake percentage of vegetables, fruit and legumes and overweight/obesity risk

(a) Fruits, (b) Vegetables, (c) Legumes. Adjusted for age, sex, physical exercise, marriage status, and education. All P for nonlinearity > 0.05.
