# Supplementary material for: Consumption of fruits, vegetables, and legumes are associated with overweight/obesity in the middle- and old-aged Chongqing residents: A case-control study
Source: Medicine (Baltimore). 2022 Jul 8;101(27):e29749. doi: 10.1097/MD.0000000000029749 (PMC9259125; doi:10.1097/MD.0000000000029749)
Supplement: Supplementary file 3 [file medi-101-e29749-s003.pdf]

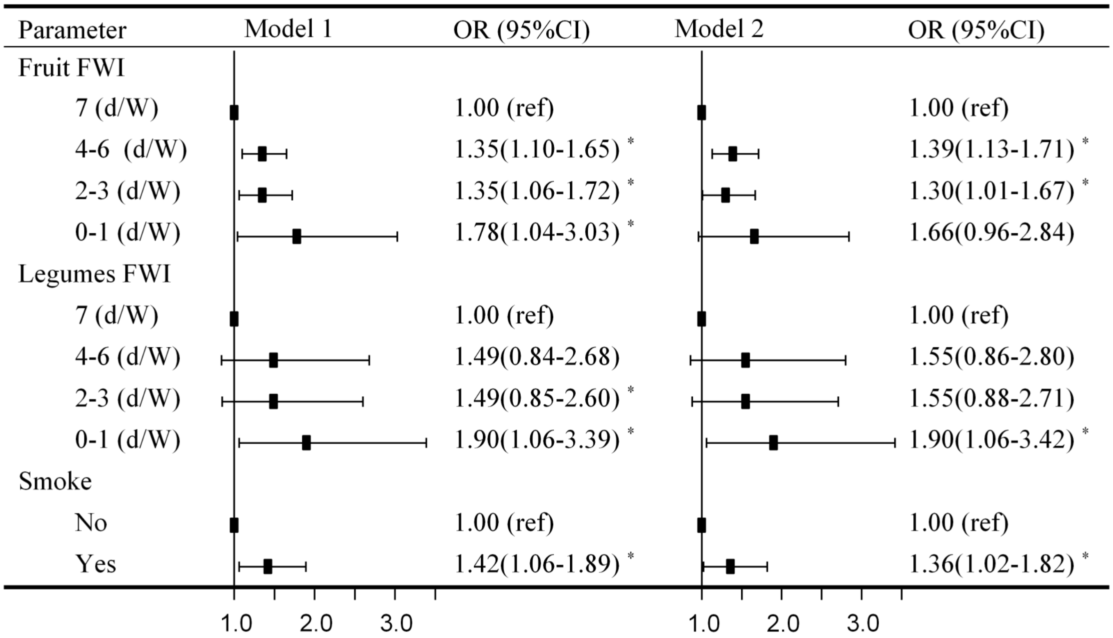

Figure S3. OR (95% CI) of overweight/obesity risk according to potential factors

Abbreviations: FWI, frequency of weekly intake; d/W, days/Week;

Model 1 adjusted for sex, age, physical exercise, marriage status, and education;

Model 2 adjusted for model 1 and disease history of hypertension, diabetes mellitus, hyperlipidemia.
