# Supplementary material for: Consumption of fruits, vegetables, and legumes are associated with overweight/obesity in the middle- and old-aged Chongqing residents: A case-control study
Source: Medicine (Baltimore). 2022 Jul 8;101(27):e29749. doi: 10.1097/MD.0000000000029749 (PMC9259125; doi:10.1097/MD.0000000000029749)
Supplement: Supplementary file 4 [file medi-101-e29749-s004.pdf]

		OR (95%CI)	*p*-value	*p*-interaction
Smoke	Vege PWI			
No	<15%	1.00 (ref)		
No	≥15%	1.18(0.92-1.51)	0.184	0.100
Yes	<15%	1.52(0.88-2.65)	0.135	
Yes	≥15%	1.57(1.08-2.29)	0.018	
Smoke	Fruits FWI			
No	2-7 d/W	1.00 (ref)		
No	0-1 d/W	1.24(0.64-2.43)	0.524	0.031
Yes	2-7 d/W	1.33(0.99-1.79)	0.062	
Yes	0-1 d/W	1.99(0.82-4.85)	0.128	
Smoke	Legumes FWI			
No	2-7 d/W	1.00 (ref)		
No	0-1 d/W	1.25(0.97-1.60)	0.082	0.008
Yes	2-7 d/W	1.36(0.99-1.88)	0.060	
Yes	0-1 d/W	1.57(0.95-2.60)	0.076	
Drink	Legumes FWI			
No	2-7 d/W	1.00 (ref)		
No	0-1 d/W	1.19(0.94-1.51)	0.145	0.046
Yes	2-7 d/W	1.15(0.77-1.71)	0.496	
Yes	0-1 d/W	2.03(1.04-3.97)	0.038	

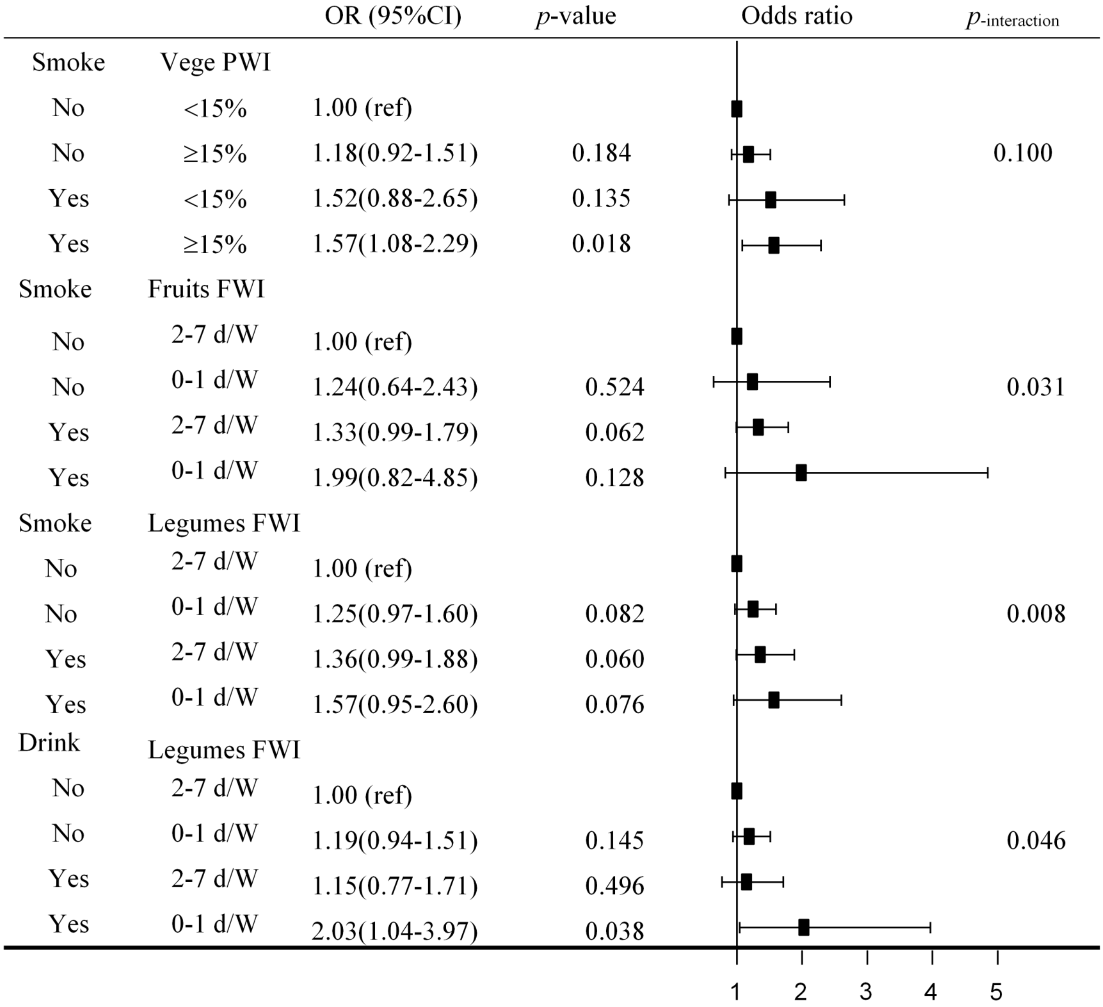

Figure S4. OR (95% CI) of overweight/obesity risk according to dietary habits and behavioral lifestyles interactions

Abbreviation: Vege, Vegetables; FWI, Frequency of Weekly Intake; PWI, Percentage of Weekly Intake; d/W, days/Week; *, $p < 0.05$.

Conditional logistic regression adjusted for sex, age, physical exercise, marriage status, education, disease history of hypertension, diabetes mellitus, and hyperlipidemia.
